# Automated MRI‐Based Classification of Parkinsonism: A Deep Learning Approach to Distinguish PD From PSP

**DOI:** 10.1111/cns.70645

**Published:** 2025-11-12

**Authors:** Xiaofei Hu, Zehong Cao, Tianbin Song, Ying Zhou, Weizhao Lu, Yingjie Zhu, Rui Hua, Dawei Peng, Feng Shi, Jie Lu

**Affiliations:** ^1^ Xuanwu Hospital Capital Medical University Beijing China; ^2^ Department of Nuclear Medicine, Southwest Hospital Third Military Medical University (Army Medical University) Chongqing China; ^3^ Department of Research and Development Shanghai United Imaging Intelligence co., Ltd. Shanghai China

**Keywords:** automated MRI‐based classification, deep learning, magnetic resonance parkinsonism index, Parkinson's disease, progressive supranuclear palsy

## Abstract

**Objective:**

Differentiating Parkinson's disease (PD) from progressive supranuclear palsy (PSP) is crucial for appropriate treatment, as each disease has distinct therapeutic requirements. The Magnetic Resonance Parkinsonism Index (MRPI) has shown promise as a diagnostic biomarker, yet manual methods introduce variability and limit its applicability. In this study, we aim to develop a fully automated algorithm for MRPI 1.0 and 2.0 calculation, and assess its ability to distinguish PD from PSP in two cohorts from different regions of China.

**Methods:**

A total of 75 PD patients and 29 PSP patients from two hospitals were enrolled. All participants underwent neurological examinations, including the MDS‐UPDRS‐III and H‐Y scale, as well as brain MRI scans. Additionally, tissue‐intensity images derived from 3D isotropic T1WI images from 2D thick slices using a deep learning (DL)‐based super‐resolution (SR) technique were aligned to a standard template followed by corresponding structural mask parcellation for measurement of MRPI 1.0 and MRPI 2.0. Subsequently, a logistic regression model was constructed to identify PD patients from PSP based on these indexes.

**Results:**

MRPI 2.0 demonstrated higher diagnostic accuracy than MRPI 1.0, with an AUC of 0.78. Additionally, the automated method showed strong linear correlations with manual assessments from an experienced radiologist, validating its reliability, and identification of PSP from PD with the average AUC of 0.85.

**Conclusion:**

The automated MRPI method improves diagnostic accuracy for differentiating PD from PSP, providing a reliable and clinically applicable tool. The integration of a super‐resolution technique to convert 2D MRI data into high‐resolution images expands the potential of MRPI as a neuroimaging biomarker.

## Graphical abstract

Using a deep learning‐based super‐resolution technique, we developed an automated method for calculating MRPI 1.0 and 2.0 from thick‐slice MRI, achieving an AUC of 0.85 in distinguishing PSP from PD. This approach enhances diagnostic accuracy, offering a reliable tool for the clinical differentiation of these parkinsonian disorders.

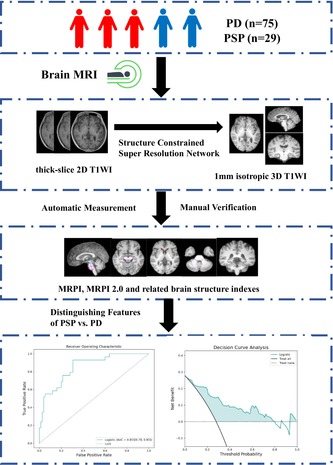

## 1 Introduction

Parkinson's disease (PD) and progressive supranuclear palsy (PSP) are two neurodegenerative disorders with overlapping symptoms. PD is characterized by abnormal aggregates of alpha‐synuclein in Lewy bodies and Lewy neurites [1], while PSP is a tauopathy with a clinical phenotype primarily characterized by parkinsonism, which can be asymmetrical and levodopa‐responsive, resembling PD [2]. The clinical differentiation between PD and PSP is particularly challenging in the early stages, as key symptoms such as supranuclear vertical gaze palsy in PSP only manifest later [3]. Differentiating between these disorders is crucial for treatment planning and prognosis, as PD and PSP require different therapeutic approaches. PSP, in particular, has a poorer prognosis and limited response to medical treatment, with interventions such as deep brain stimulation being ineffective. Current PSP management focuses on symptomatic treatment including physical therapy for mobility and fall prevention, speech therapy for dysarthria and dysphagia, and certain medications such as botulinum toxin for dystonia, though the therapeutic response remains limited compared to PD [4]. This necessitates a simple and accurate method for differentiating between these disorders.

Neuroimaging biomarkers, particularly from MRI, offer valuable diagnostic insights. The Magnetic Resonance Parkinsonism Index (MRPI), calculated as the ratio of the pons area to midbrain area multiplied by the ratio of the middle cerebellar peduncle width to the superior cerebellar peduncle width, has proven to be a potent biomarker for distinguishing PSP from PD. Studies have reported MRPI's sensitivity between 70% and 100% and specificity from 43% to 100% [5, 6]. MRPI 2.0, which includes the third ventricle width in its calculation, has shown improved performance in distinguishing PSP‐P from PD in the early stages [7, 8]. However, past studies have relied on manual measurements, introducing potential for inter‐rater discrepancies and making it challenging to compare results between different cohorts and sites.

Recent developments include automated procedures for computing MRPI, but they have focused only on MRPI or MRPI 2.0, using high‐resolution scans at specialized centers [9, 10]. This approach incurs high costs and lengthy acquisition times. Therefore, developing a screening model that can achieve comparable diagnostic accuracy with shorter scan protocols would represent significant progress for clinical practice.

This study aimed to develop a fully automated algorithm to calculate MRPI 1.0 and 2.0, distinguishing PSP from PD across two cohorts from different regions in China. It also explored a deep learning (DL)‐based super‐resolution (SR) technique to convert thick‐slice MRI images into high‐resolution isotropic data, facilitating advanced morphometric analysis. The study further assessed the repeatability and reproducibility of the volumetry, and explored machine learning methods to train from original indicators to improve classification performance.

## 2 Materials and Methods

### 2.1 Patients

This study involved 75 patients with idiopathic Parkinson's disease (PD) and 29 patients with progressive supranuclear palsy (PSP), enrolled from two centers: the Movement Disorder Center at Southwest Hospital and Xuanwu Hospital. Diagnoses of PD and PSP were made by movement disorder specialists using international diagnostic criteria [11, 12]. Patients diagnosed with PSP before 2017 were reclassified according to recent Movement Disorder Society (MDS) diagnostic criteria for probable PSP [11].

All patients underwent neurological examinations, including the MDS‐sponsored revision of the Unified Parkinson's Disease Rating Scale part III (MDS‐UPDRS‐III) in the off‐state, and the Hoehn and Yahr (H‐Y) rating scale [13]. Exclusion criteria included age < 40 years, clinical features suggestive of other diseases, and MRI abnormalities such as lacunar infarctions or subcortical vascular lesions with diffuse periventricular signal alterations.

All study procedures were approved by an institutional review board, and each recruitment site received approval from its institutional review board or ethics committee. Written informed consent was obtained from all individuals, in accordance with the Declaration of Helsinki, for the use of their medical records for research purposes. This study utilized a retrospective analysis design, where MRI scans and clinical evaluations were performed as part of routine clinical care following standardized diagnostic protocols.

### 2.2 MRI Protocol

All patients underwent brain MRI using either 3 T or 1.5 T scanners, including Siemens scanners at Southwest Hospital, and Philips and GE scanners at Xuanwu Hospital. The protocol included both routine 2D thick‐slice T1‐weighted images (5–6.5 mm slice thickness) and high‐resolution 3D T1‐weighted sequences within a single scanning session, with the imaging parameters summarized in Table 1. The 2D thick‐slice acquisition required approximately 3–5 min, while the 3D high‐resolution sequences required 8–12 min of scan time. The protocol included a T1‐weighted volumetric image, with the imaging parameters summarized in Table 1.

**TABLE 1:** T1‐weighted sequence parameters for each MRI site.

Center	Scanner manufacture	Scanner model	Cases	Magnetic field (T)	Slice thickness (mm)	Spacing between slices	TE (ms)	TR (ms)
Southwest Hospital	SIEMENS	Sonata	28	1.5	5	6.5	4.88	230
		TrioTim	40	3	5	6.5	2.78	200
Xuanwu Hospital	Philips	Ingenia	17	3	5	6.2	2.302	133
	GE	SIGNA Pioneer	19	3	5	6.5	2.1	120

### 2.3 Methods

**FIGURE 1:**
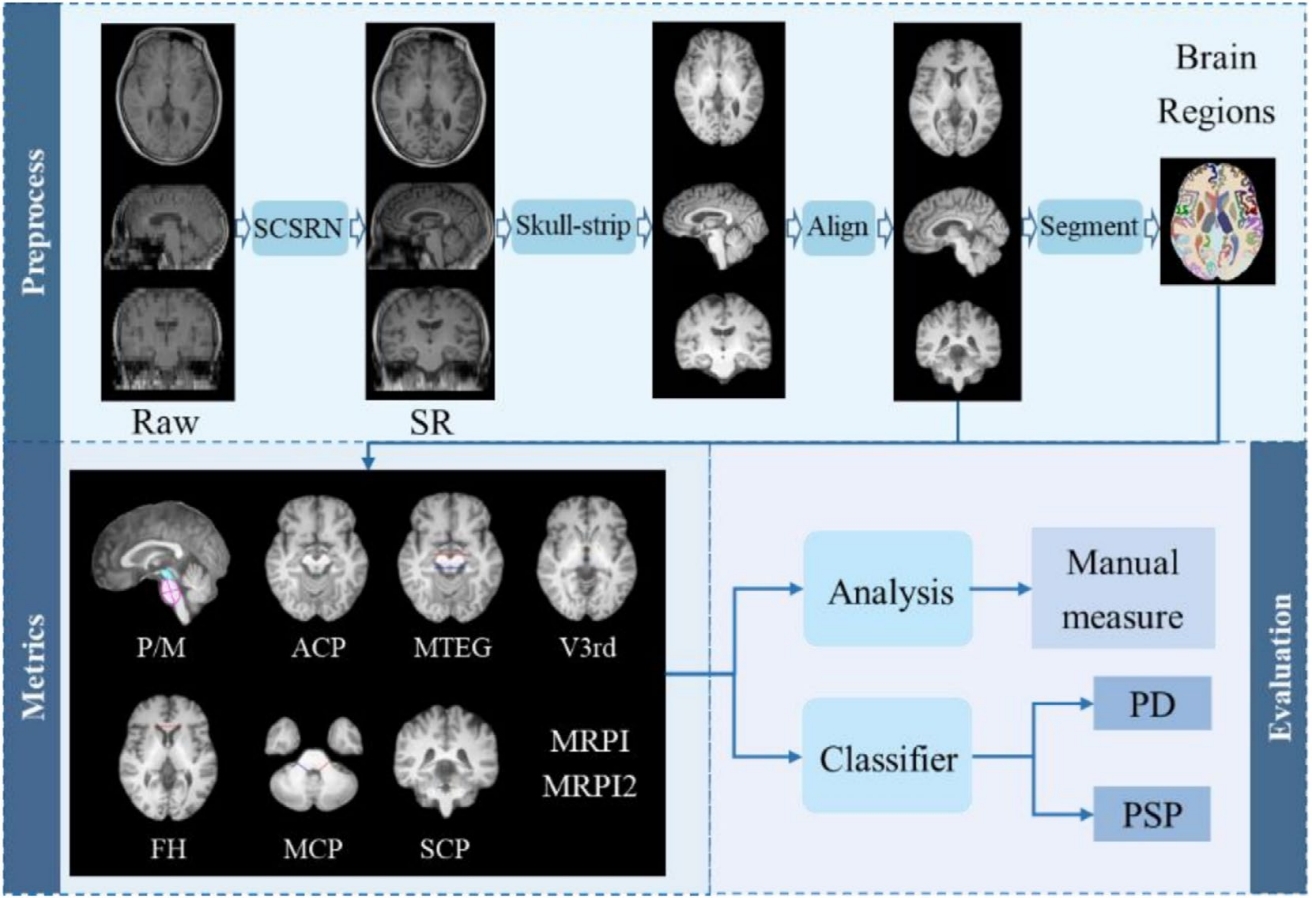
The framework of automatic measurement about PD‐relation indexes.

A comprehensive framework was developed for the automatic measurement of MRPI and other PD‐related indexes through the following steps: (1) Image enhancement: The resolution of diagnostic 2D T1‐weighted images (T1WI) was reconstructed to 1 mm isotropic 3D T1WI using a novel deep learning (DL) algorithm in our previous work, Structure Constrained Super Resolution Network (SCSRN) [14], which has demonstrated high performance, achieving a PSNR of 27.04 ± 0.932 and an SSIM of 0.9958 ± 0.0009 on the main testing dataset and high consistency with native 3D MR images, ensuring the preservation of anatomical structures required for accurate MRPI measurements. (2) Alignment and standardization: To eliminate the impact of head rotation, skull‐stripped images were rigidly aligned to MNI‐152 standard spacing in the ANTs package [15]. (3) Structural masks generation: Images without skulls were segmented into 109 brain regions of interest (ROI) using an in‐house tool [16]. (4) Index measurement and classification: MRPI and MRPI 2.0 were taken from the ROI masks, and these indexes were employed to distinguish PSP patients from PD patients (as seen in Figure 1).

### 2.4 Procedure

Briefly, with MRPI, the majority of the relevant measurement principles strictly adhere to the approach previously presented in the literature [9, 10, 17], and all metrics are conducted on corresponding 2D planes shown in Figure 1. Considering the relationship between related indexes and anatomy, brain structure masks, segmented from super‐resolution reconstructions of clinical routine T1 images, were utilized to automatically identify these regions. Notably, for the reliability and sophistication of auto‐computation, some metrics have been tailored to the measurement details provided in Supporting Information S1 (see Figure S1 for a detailed illustration of measurement procedures).

To validate the automated measurement method, 14 cases (10 PD, 4 PSP) were randomly selected from the dataset. An experienced radiologist evaluated the measurements, which were used as a reference for these cases.

### 2.5 Statistical Analysis

Statistical analyses were performed using Python 3.7 libraries, including scipy (ver. 1.7.3), numpy (ver. 1.19.0), and pandas (ver. 1.1.5). The Kruskal‐Wallis test was used to assess differences between PSP and PD measurement indexes. The Kolmogorov–Smirnov test evaluated normality, determining if parametric or nonparametric tests were appropriate. Spearman's method calculated coefficients between automated and manual indexes. All analyses considered the MRI data as a single sample, without factoring in the study site. Cross‐validation employed stratified sampling to ensure that the proportion of PD and PSP cases was preserved in each fold, maintaining the class imbalance to reflect real‐world data distribution.

## 3 Result

The final cohort included 67 patients from the Southwest Hospital (16 with PSP, 51 with PD), and 37 patients from Xuanwu Hospital (13 with PSP, 24 with PD). The demographic, clinical, and imaging data of patients from both cohorts are summarized in Table 2. In both cohorts, PSP patients were significantly older than PD patients; thus, all analyses were corrected for age at examination. Patients with PSP had similar disease duration but higher disease severity compared to PD patients (Table 2).

**TABLE 2:** Demographic data of study participants.

	PD	PSP	*p*
Sex (M/F)	49/36	19/10	
Age (y, mean ± SD)	62.5 ± 9.1	70.5 ± 6.1	0.14
Disease duration (y, mean ± SD)	5.7 ± 4.3	4.4 ± 2.6	0.58
MDS‐UPDRS‐III score (median, range)	23.6 ± 11.6	27.2 ± 15.3	0.68
H‐Y score (median, range)	2.3 ± 0.7	3.4 ± 0.7	0.03*

*Note:* Significance of * indicates statistical significance of the *p*‐values when *p < 0.05 vs. PD (Kruskal‐Wallis test).

Abbreviations: PD, Parkinson's disease; PSP, progressive supranuclear palsy.

### 3.1 Evaluation of Indexes Between AI and Radiologist

**FIGURE 2:**
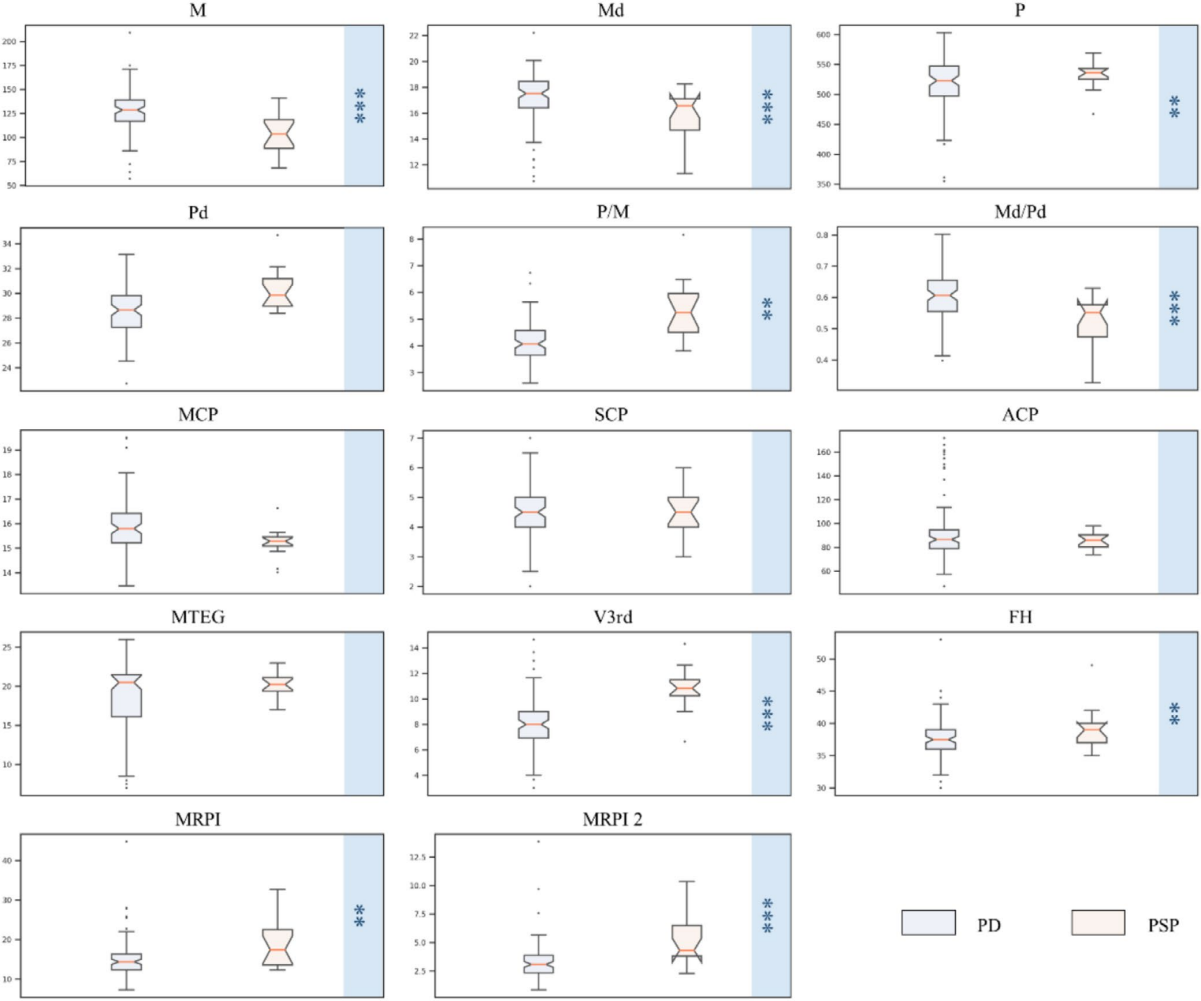
Measurement results of our method for the PD and PSP including 104 subjects. Kruskal‐Wallis Test, **p < 0.01, ***p < 0.001.

Figure 2 presents the measurement results from our method. As shown in Figure 2, compared to PD, PSP patients exhibit significant midbrain atrophy and third ventricle dilation. Kruskal‐Wallis tests revealed significant differences between PD and PSP groups across various indicators related to the midbrain, pons, and third ventricle, as well as maximum frontal horn width (*p* < 0.01).

**FIGURE 3:**
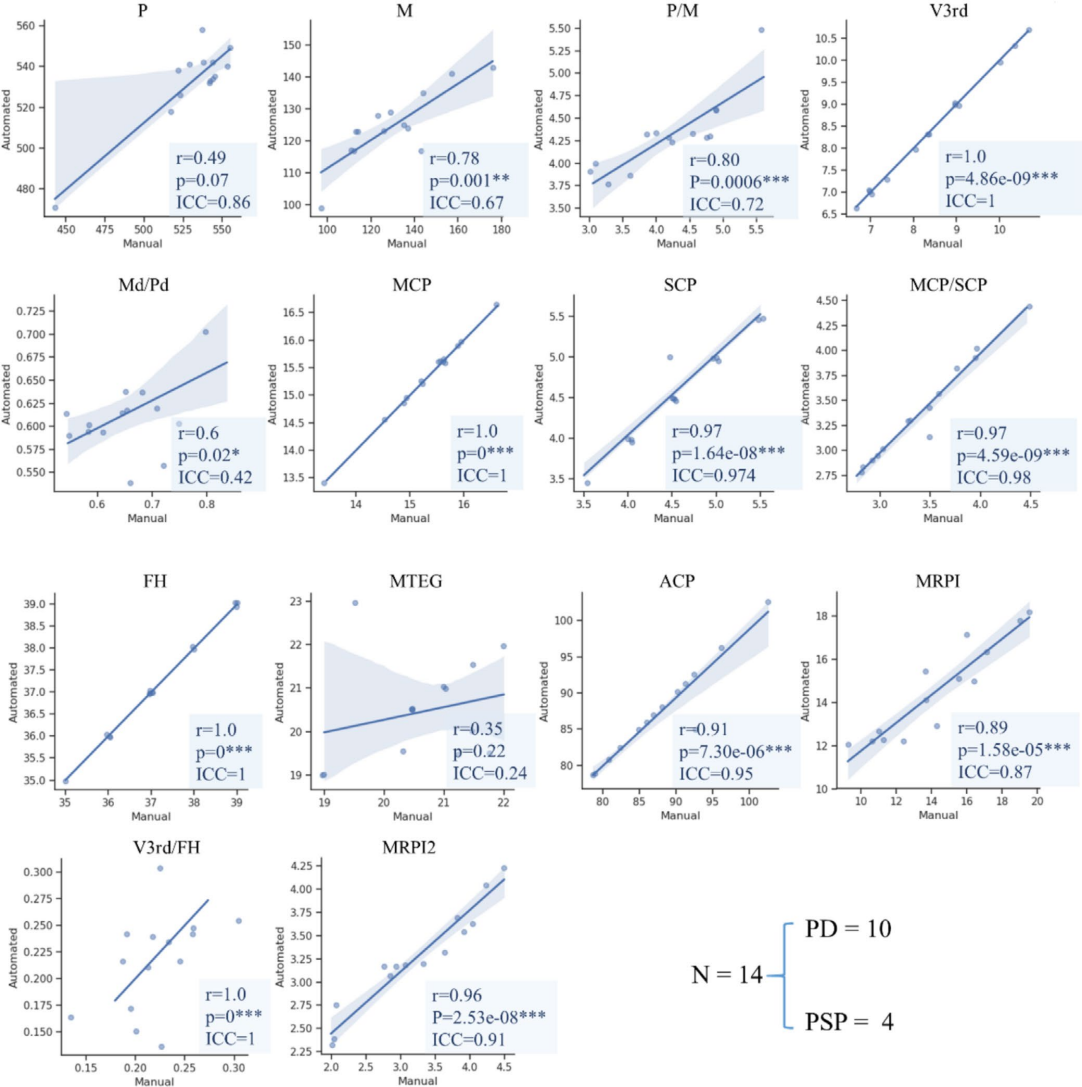
Correlations between automatic and manual metrics about Magnetic Resonance Parkinsonism Index 2.0 (MRPI 2.0) values in a subgroup of 14 participants. SIgnificance of *,**,*** indicates the spearman's correlation coefficients when *p < 0.05, **p < 0.01, ***p < 0.001 respectively.

To estimate the consistency between manual and automatic results, we compared the results from an experienced radiologist with those calculated by our automated method for 14 randomly selected cases. Figure 3 illustrates the correlation between these results. Linear correlations were found among most indicators.

### 3.2 Differentiation of PD and PSP Cases of Proposed Method

**FIGURE 4:**
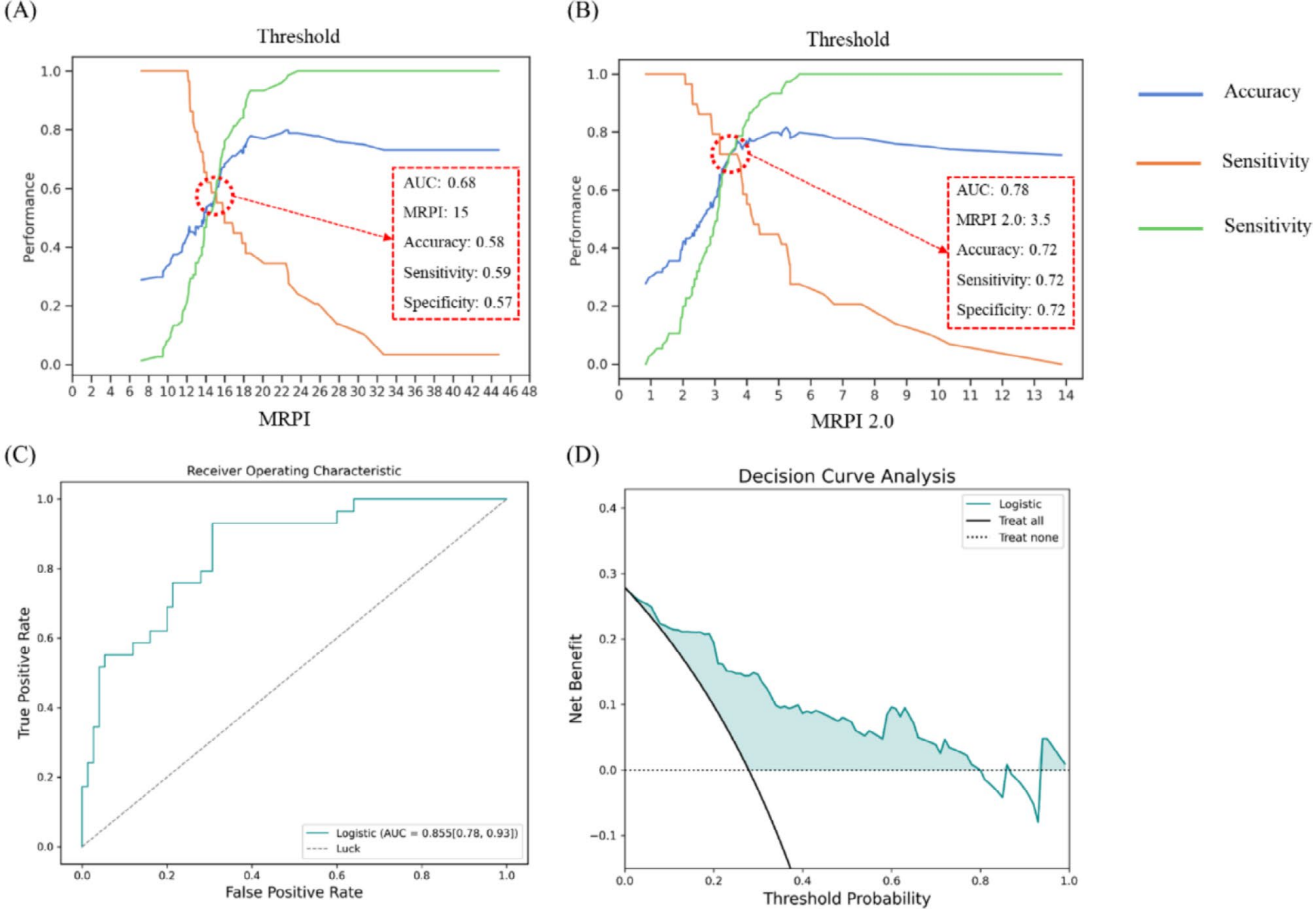
Discrimination of PD and PSP with different MRPI and MRPI 2.0 thresholds shown in (A) and (B) at the first line. (C) and (D) are respectively the ROC curve and DCA of classification tasks about PD/PSP with the logistic model from AI results in 104 cases. Original Metrics: 16 (P, M, Pd, Md, MCP, SCP, ACP, MTEG, V3rd, FH, P/M, MCP/SCP, Md/Pd, V3rd/FH, MRPI, MRPI 2.0).

We directly distinguished between PD and PSP using MRPI and MRPI 2.0. MRPI 2.0 demonstrated better discrimination between the two disorders, achieving an AUC of 0.78. With the curves shown in Figure 4A,B, after balancing the values of accuracy, sensitivity, and specificity, we recommend a threshold of 15 for MRPI and 3.5 for MRPI 2.0 to distinguish PSP from PD.

To further evaluate the performance of our method, we propose a procedure for diagnosing PSP from classical PD patients:

1. We obtain the super‐resolution image from raw thickness data and corresponding brain segmentation results using an in‐house toolbox.
2. PD‐related indexes are measured from segmented brain regions using our method, yielding 16 features (P: Area of pons, M: Area of midbrain, Pd: Diameter of pons, Md: Diameter of midbrain, MCP: Thickness of the middle cerebellar peduncles, SCP: Thickness of the superior cerebellar peduncles, ACP: Angle of cerebral peduncles, MTEG: Diameter of the midbrain tegmentum, V3rd: Width of the third ventricle, FH: Frontal horn distance of the lateral ventricles, P/M, MCP/SCP, Md/Pd, V3rd/FH, MRPI, MRPI 2.0).
3. A Logistic Regression (LR) classifier with default parameters classifies patients as PSP or PD (PSP: 29, PD: 75). We do not use sophisticated classification and feature selection methods, as the main goal is to evaluate the reliability of the automated MRPI measurement method. The choice of logistic regression was validated through comparative analysis with other machine learning algorithms, as detailed in Table S1.
4. Five‐fold cross‐validation demonstrated robust performance with consistent AUC values across folds (0.80–0.96) and an overall accuracy of 80% (Table 3). The average AUC for differentiating PSP and PD is 0.85, and the ROC curve is shown in Figure 4C,D. Feature importance analysis revealed that MRPI 2.0, midbrain area, and third ventricle width were the most discriminative features for PSP versus PD classification (Figure S3).

**TABLE 3:** Metrics of 5‐fold validation for classification of PD‐PSP.

Metrics	Fold‐1	Fold‐2	Fold‐3	Fold‐4	Fold‐5	Total
AUC	0.84	0.80	0.96	0.87	0.87	0.85
Accuracy	0.86	0.67	0.81	0.76	0.90	0.80
Sensitivity	0.50	0.33	0.67	0.67	0.80	0.59
Specificity	1.00	0.80	0.87	0.80	0.93	0.88

## 4 Discussion

This study reports the development of a fully automated algorithm for the measurement of the Magnetic Resonance Parkinsonism Index (MRPI) 1.0 and MRPI 2.0, two powerful neuroimaging biomarkers that can help distinguish Parkinson's disease (PD) from progressive supranuclear palsy (PSP). The main findings are: (1) The proposed automated method demonstrated high reliability, with strong correlations between the automatically calculated MRPI values and those obtained through manual assessments by an experienced radiologist. (2) MRPI 2.0 showed superior performance compared to MRPI 1.0 in differentiating PD from PSP, achieving an average AUC of 0.85 in a classification task. (3) Importantly, this automated approach enables the utilization of routine diagnostic 2D T1‐weighted MRI data, which are readily available in clinical settings, for advanced morphometric analyses.

The diagnostic performance of the MRPI measures demonstrated in this study is consistent with and builds upon recent literature on this topic. Prior studies have reported that the original MRPI exhibited a wide range of sensitivity (70%–80%) and specificity (43%–60%) in distinguishing PD from PSP patients [5, 6]. The improved diagnostic accuracy of MRPI 2.0 observed in the current work aligns with more recent findings that this refined index, which incorporates the third ventricle width, can better capture the characteristic brain atrophy patterns that differentiate these two parkinsonian disorders, especially in the earlier stages [7, 8].

The superior performance of MRPI 2.0 underscores its potential as a valuable diagnostic tool to complement clinical assessments, which can be challenging to differentiate PD and PSP, particularly in the initial phases of the diseases [8]. Early and accurate diagnosis is pivotal for guiding appropriate treatment strategies and management, given the stark differences in prognosis and therapeutic approaches between PD and atypical parkinsonian disorders like PSP. The reliable automated measurement approach developed in the current study further enhances the clinical utility of MRPI, addressing limitations of prior manual techniques that introduced potential biases and inconsistencies across different clinical settings [9, 18].

A notable strength of our study lies in the diverse nature of the dataset, which was acquired from two different centers, the Movement Disorder Center at Southwest Hospital and Xuanwu Hospital, using multiple MRI scanners (3 T and 1.5 T) from various manufacturers (Siemens, Philips, and GE). This heterogeneity in data acquisition enhances the robustness and generalizability of our findings, as it reflects the real‐world variability encountered in clinical settings. Furthermore, the ability to reliably compute MRPI from data acquired on different scanners and field strengths broadens the potential patient pool eligible for this diagnostic biomarker. This is especially relevant in resource‐limited settings or regions where access to advanced imaging facilities may be limited.

One of the key innovative aspects of our study is the utilization of a deep learning‐based super‐resolution technique to convert thick‐slice 2D T1WI into high‐resolution 3D T1WI isotropic data. This approach addresses a significant limitation in most clinical settings, where acquisition of high‐resolution 3D T1WI is often impractical due to time constraints and potential patient motion artifacts. This novel approach represents a significant departure from prior work [14]. By enabling the use of clinically acquired 2D MRI data, this super‐resolution method greatly expands the potential applicability of MRPI and other quantitative neuroimaging biomarkers. Conventional interpolation methods for converting 2D T1WI to 3D isotropic data often result in blurring and loss of fine structural details, limiting their utility for advanced morphometric analysis. Our DL‐based SR technique leverages the power of deep neural networks to learn and reconstruct high‐frequency information from low‐resolution images, effectively enhancing spatial resolution and preserving structural details [19, 20]. The deep learning‐based reconstruction approach developed here effectively bridges this gap, transforming Diagnostic MRI scan data into a format suitable for comprehensive brain structure analyses [19]. Furthermore, the automated nature of the super‐resolution technique not only reduces scan times and patient burden but also mitigates the variability and potential biases introduced by manual image processing, further strengthening the reliability and clinical applicability of the MRPI‐based diagnostic approach. The automated MRPI measurement pipeline has been integrated into the uAI Brain Health Evaluation Software (registered software copyright in China), enabling practical clinical deployment with PACS system integration (Figure S2).

The proposed automated MRPI measurement method not only demonstrates excellent diagnostic performance in distinguishing PSP from PD but also offers standardization, efficiency, and reproducibility, laying the foundation for integrating MRPI as a biomarker into clinical practice. The automated MRPI method can be easily incorporated into existing clinical imaging workflows, without requiring additional scan sequences or prolonged scan times, by directly taking input from routine 2D T1‐weighted images. Overall, the automated MRPI measurement method provides a feasible, efficient, and standardized tool for clinical decision‐making, with the potential to promote the broader application of MRPI biomarkers in the diagnosis and management of parkinsonian spectrum disorders.

This study includes several limitations. First, we utilized 75 PD patients and 29 PSP patients, presenting a significant imbalance. Such an imbalance can lead to a biased model that favors the majority class, in this case, PD, which may restrict the accuracy and sensitivity in identifying PSP cases—a condition notably rarer than PD. This rarity also influenced our decision not to further subclassify PSP cases, aligning our study more closely with real‐world clinical scenarios where rapid differentiation between PD and PSP is crucial for advancing clinical management processes. Moreover, our total sample size was relatively small, which can reduce confidence in the results and increase the margin of error. Nevertheless, we have attempted to mitigate this limitation through methodological rigor, such as the use of a training cohort and an independent testing cohort, and by comparing the results from an experienced radiologist with those calculated by our automated method for 14 randomly selected cases. Additionally, the generalizability of our findings could be impacted by the regional characteristics of the cohorts, both sourced from China. Future studies should validate these findings in larger, more diverse patient populations and consider the integration of more advanced machine learning models to further enhance the classification performance. Additionally, further development and testing to streamline the automated MRPI measurement process would be valuable, making it more accessible for real‐world clinical practice, particularly in differentiating parkinsonian disorders [9, 18]. Longitudinal studies tracking the progression of PD and PSP using automated MRPI measures could also provide important insights to aid in treatment and management strategies [8].

In conclusion, this study presents an important step forward in the development of automated, reliable, and clinically applicable neuroimaging biomarkers for the differential diagnosis of parkinsonian disorders. The ability to leverage routine diagnostic MRI data for advanced morphometric analyses, as demonstrated here, could significantly expand the utility of these biomarkers and improve the timely detection and management of conditions like PSP. Ongoing research to refine and validate these automated methods, as well as explore their longitudinal application, will be crucial to realizing the full potential of MRI‐based tools in the clinical care of patients with parkinsonian syndromes.

Nevertheless, there are limitations that should be acknowledged. Most notably, the sample size for the PSP group was relatively small (*n* = 29) compared to the PD group (*n* = 75), resulting in a substantial class imbalance. This disparity may have introduced bias into the classification model, affecting its robustness and, in particular, reducing the sensitivity for PSP detection, as reflected by the lower sensitivity observed in our results. In future research, we plan to validate the robustness and classification ability of our automated measurement method using larger and more diverse datasets. By expanding both the overall sample size and the representation of the PSP group, we aim to enhance the reliability and clinical relevance of our results. Additional limitations include the lack of PSP subtype classification (Richardson syndrome vs. PSP‐parkinsonism), which may have affected classification performance as different PSP subtypes exhibit varying patterns of brain atrophy. The cross‐validation employed stratified sampling to maintain class proportions, but no additional corrective measures for class imbalance were applied beyond stratification. Furthermore, while our study included data from multiple scanners, no specific harmonization techniques were applied beyond standard preprocessing, which may limit cross‐scanner reproducibility. The compatibility of our super‐resolution approach with shorter scan protocols or lower field strengths in resource‐limited settings has not been systematically validated and remains to be established in future studies.

## Author Contributions

F.S. and J.L. conceived the study. X.H. and Z.C. collected and analyzed the data. X.H., T.S., Y.Z., and W.L. wrote the manuscript. Y.Z., R.H., and D.P. revised and reviewed the manuscript. All authors contributed to the article and approved the submitted version.

## Ethics Statement

This retrospective imaging study used de‐identified data collected during routine clinical care at Southwest Hospital and Xuanwu Hospital. The protocol was reviewed and approved by the [Research Ethics Committee of Southwest Hospital, No.: (B)KY2023026; Research Ethics Committee of Xuanwu Hospital, No.: (2023)044].

## Conflicts of Interest

The authors declare no conflicts of interest.

## Supporting information


**Figure S1:** The diagram of metrics on the corresponding slice. In the first row, from left to right, are the area of midbrain and pons (M, P), width of the third ventricle (3rdV), the maximum frontal horns width (FH), and the middle cerebellar peduncles (MCP). The second row, are the fitted ellipses of midbrain and pons, ACP, METG, and SCP.
**Figure S2:** Schematic illustration of the user interface of the uAI Brain Health Evaluation Software.
**Figure S3:** Quantitative analysis of feature importance based on the values of the average coefficients from the five‐fold cross‐validated logistic regression models.
**Table S1:** Five folds comparative analysis of logistic regression, random forest, and support vector machine (SVM) classifiers.

## Data Availability

The data that support the findings of this study are available on request from the corresponding author. The data are not publicly available due to privacy or ethical restrictions.
