# Supplementary material for: Automated MRI‐Based Classification of Parkinsonism: A Deep Learning Approach to Distinguish PD From PSP
Source: CNS Neurosci Ther. 2025 Nov 12;31(11):e70645. doi: 10.1111/cns.70645 (PMC12611708; doi:10.1111/cns.70645)
Supplement: Supplementary file 1 — Figure S1: The diagram of metrics on the corresponding slice. In the first row, from left to right, are the area of midbrain and pons (M, P), width of the third ventricle (3rdV), the maximum frontal horns width (FH), and the middle cerebellar peduncles (MCP). The second row, are the fitted ellipses of midbrain and pons, ACP, METG, and SCP. Figure S2: Schematic illustration of the user interface of the uAI Brain Health Evaluation Software. Figure S3: Quantitative analysis of feature importance based on the values of the average coefficients from the five‐fold cross‐validated logistic regression models. Table S1: Five folds comparative analysis of logistic regression, random forest, and support vector machine (SVM) classifiers. [file CNS-31-e70645-s001.docx]

Automated MRI-Based Classification of Parkinsonism: A Deep Learning Approach to Distinguish PD from PSP

Xiaofei Hu^1，2#^, Zehong Cao^3#^, Tianbin Song^1^, Ying Zhou^1^, Weizhao Lu^1^, Yingjie Zhu^2^, Rui Hua^3^, Dawei Peng^3^, Feng Shi^3*^, Jie Lu^1*^

**Detailed Methods for Customized Metrics**

In this section, we outline the specific procedures for deriving customized metrics from the MRI data, which have been adapted to address unique aspects of our study's requirements. Each metric was carefully developed to ensure accuracy in capturing relevant neuroanatomical features (Supplementary Figure 1), and their computation was tailored to the clinical context of differentiating Parkinson's disease (PD) from progressive supranuclear palsy (PSP). Here are the detailed steps for each customized metric:

**Area of midbrain and pons.** Firstly, we align the tissue-intensity image and structure mask to a standard MRI template in a rigid way^1^ to adjust the brain orientation and extract the mid-sagittal slice. Based on the structure mask, we directly counted the number of pixel points in the mid-brain and pons respectively as the area of corresponding regions at the medial sagittal plane. Inevitably, when the midbrain is too close to the upper edge of the tegmentum, it causes our automatic brain region segmentation to be unable to separate them perfectly and completely. To address this situation, we used the watershed algorithm to perform a secondary fine segmentation of these two regions.

**Diameter of midbrain and pons.** In Neurology of 2013, Luke A. Massey proposed an evaluation index of the intrinsic elliptical aspect ratio fitted to the midbrain and pontine portion was used to discriminate between patients with PSP^2^. With the pathological report as the ground truth, this ratio proved to have high sensitivity and specificity in differentiating PSP from other subtypes of PD. For the sake of comprehensiveness of the assessment, we likewise introduced the measurement of this index. On the basis of the structural segmentation map of the brain, we extracted the part of the brain mask corresponding to the midbrain and pons in the median sagittal plane. And then, we calculated the ratio of the length diameter from the two fitted inline ellipses derived by contours of these two regions separately using opencv package^3^.

**Middle cerebellar peduncles.** According to related papers^4^, the middle cerebellar peduncles (MCP) generally is acquired from the sagittal paramedian slice of T1-weighted image defined as the mean of thickness measure of MCP at its best exposure. Due to the best exposure is too difficult to quantity and the MRI images in this experiment are super-resolution reconstructed from anisotropic data that $0.3\times0.3\mathrm{cm}^{2}$ resolution in plane and 6.5 cm thickness, we decided to measure the MCP at the axial slice where the diameter of the pons is largest along the sagittal direction, which would be more robustness for our special images. At the plane of our defined MCP, the outline of the fourth ventricle is found and considered as the inner contour towards which we calculate the MCP. Then, the outer contour of the combined cerebellar and pontine region at the same level was located, and served as the outer contour for calculating the MCP. In the meantime, the left and right sides of the inner and outer contours were divided according to the position where the median sagittal position was placed. Then, the minimum distance between the inner and outer contours of the left and right sides was calculated separately, and the mean value was taken as the MCP value obtained from our measurement.

**Superior cerebellar peduncles.** As reference papers^5,6^, the superior cerebellar peduncles (SCP) generally is acquired from several consecutive slices of coronal oblique reconstruction of the same isotropic sequence at the level of separation of the inferior colliculi. Since our segmentation mask excluded independent inferior colliculi structures, coupled with the tiny structure of the SCP, we selected to aid localization by including the location of the pons to avoid inaccurate positioning due to segmentation errors: at layers of the SCP, the starting slice of the pons that junction left and right and spanned the median sagittal plane was further defined as the measurement level of the SCP. In measuring the SCP, we considered the SCP region and the pons as a whole, and obtained the average height of the highest points on the left and right sides of this region at the coronal level that we picked, as well as the height of the lowest point of the internal contour of this region, and calculated the height at the upper third of the difference between these two heights. With this height as the reference plane, the two distances between the left and right inner and outer contours of the above-mentioned area and their intersection points are calculated, and the average value is the SCP measurement we defined.

**Angle of Cerebral Peduncles (ACP).** As reference^7^, ACP was defined as the angle formed by the posterior half of the cerebral peduncles at the level of the mammillary bodies or immediately below. In the axial plane, we first locate the slice where the mammillary bodies are visible by obtaining the mask of the mammillary bodies from the structural image and recording the corresponding slice numbers. Then, within these slices, we identify the slice where the cerebral aqueduct begins to appear as the measurement plane for the angle of the cerebral peduncles (ACP). Since the ACP is the angle formed by the intersection of the tangents to the anterior medial border of the cerebral peduncles, which are located in the area where the cerebral peduncles attach to the midbrain, we directly obtain the midbrain mask and contour on the measurement plane. We then use logistic regression to identify the two tangents to the anterior medial border of the cerebral peduncles and calculate their intersection angle as the ACP measurement value.

**Diameter of the midbrain tegmentum**: At the level of ACP measurement, the diameter of the tegmentum of midbrain (MTEG) was measured as the perpendicular distance between the center point of the midbrain aqueduct and the line connecting the anterior border of the midbrain on both sides. On the axial slice where the ACP was measured, the highest points on both sides of the midbrain were obtained by fitting the outer contour of the midbrain (with the mid-sagittal plane used as the dividing line), and then a line was drawn connecting these points. A parallel line passing through the center of the midbrain aqueduct was drawn, and the distance between the two lines was measured as the MTEG.

**Frontal horn distance:** To expand on this measurement MRPI, a new version called MRPI 2.0 has been introduced, which includes additional measurements of the third ventricle width and the frontal horn distance^8^. Studies have demonstrated that MRPI 2.0 provides superior accuracy compared to the original MRPI metric in distinguishing patients with progressive supranuclear palsy (PSP) from those with early-stage Parkinson’s disease (PD). To determine the "Frontal Horn distance (FH)", the axial view with the maximal dilation was assessed and the widest distance between the left and right sides was measured. In order to identify the level with the maximal length of the frontal horn, we started from the level where the third ventricle appeared and calculated the frontal horn distance layer by layer. Finally, we measured the width of the frontal horn at the level with the maximal length, which was considered as the measurement value of FH.

**The 3rd ventricle width.** Generally, to measure the width of the third ventricle (V3rd), three different measurements were taken of the maximum linear distance between the lateral borders on an axial slice generated at the level of both the anterior and posterior commissures. The average of these three measurements was then calculated. To improve the efficiency of automated measurements, we first used a brain region segmentation map to select the axial slice with the largest third ventricle area. Then, at the largest slice, we measured the width of the third ventricle at the midpoint and at the upper and lower quartiles of the third ventricle region. The average of these three measurements was used as our measurement value for the width of the third ventricle.


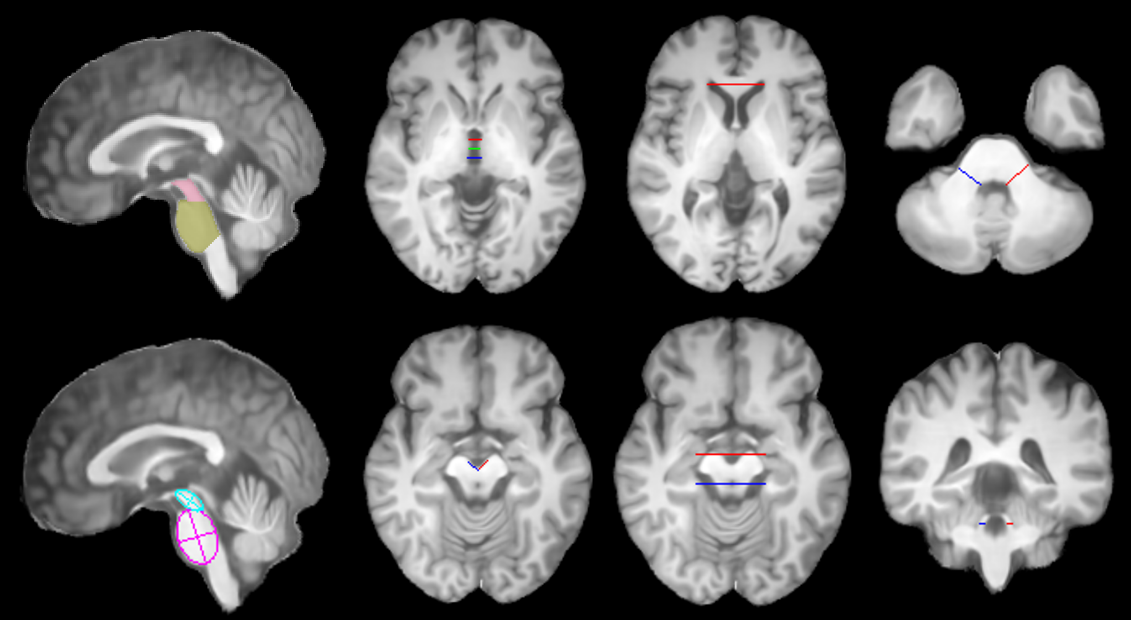


Supplementary Figure 1. The diagram of metrics on the corresponding slice. In the first row, from left to right, are the area of midbrain and pons (M, P), width of the third ventricle (3^rd^V), the maximum frontal horns width (FH), and the middle cerebellar peduncles (MCP).The second row, are the fitted ellipses of midbrain and pons , ACP, METG, and SCP.

**Brain region segmentation.** Briefly, preprocessing steps included skull stripping, bias correction, and resampling of all images to 1 mm isotropic resolution. Subsequently, the T1-weighted images were parcellated into 109 regions of interest (ROIs), with cortical surface reconstruction performed according to the DK atlas [9], based on a pre-trained, cascaded VB-Net model. Both the model and the normative reference range were established using 1,800 T1-weighted images from the publicly available Consortium for Reliability and Reproducibility (CoRR) dataset [10] and the Chinese Brain Molecular and Functional Mapping (CBMFM) project [11]. The segmentation model integrates coarse localization and segmentation refinement steps, and has previously demonstrated effectiveness in various medical image segmentation tasks, including brain tumors [12] and thoracic organs [13].

**Clinical practicality.** The super-resolution reconstruction on the main test dataset takes approximately 100 seconds per case on an NVIDIA GPU with 12 GB memory, and the total end-to-end processing time, including MRPI calculation, is within 120 seconds per case. This efficiency is acceptable for clinical workflow and minimizes delays for routine use. The entire pipeline has been fully integrated into the uAI Brain Health Evaluation Software, which supports seamless connection to hospital PACS systems as shown in Supplementary Figure 2. The system can automatically retrieve imaging data and generate structured MRPI reports with minimal user intervention, making it practical for daily clinical application. In terms of hardware, the minimum requirement is satisfied by a workstation equipped with NVIDIA RTX 4000 8GB GPU, which is a configuration commonly available in radiology departments.


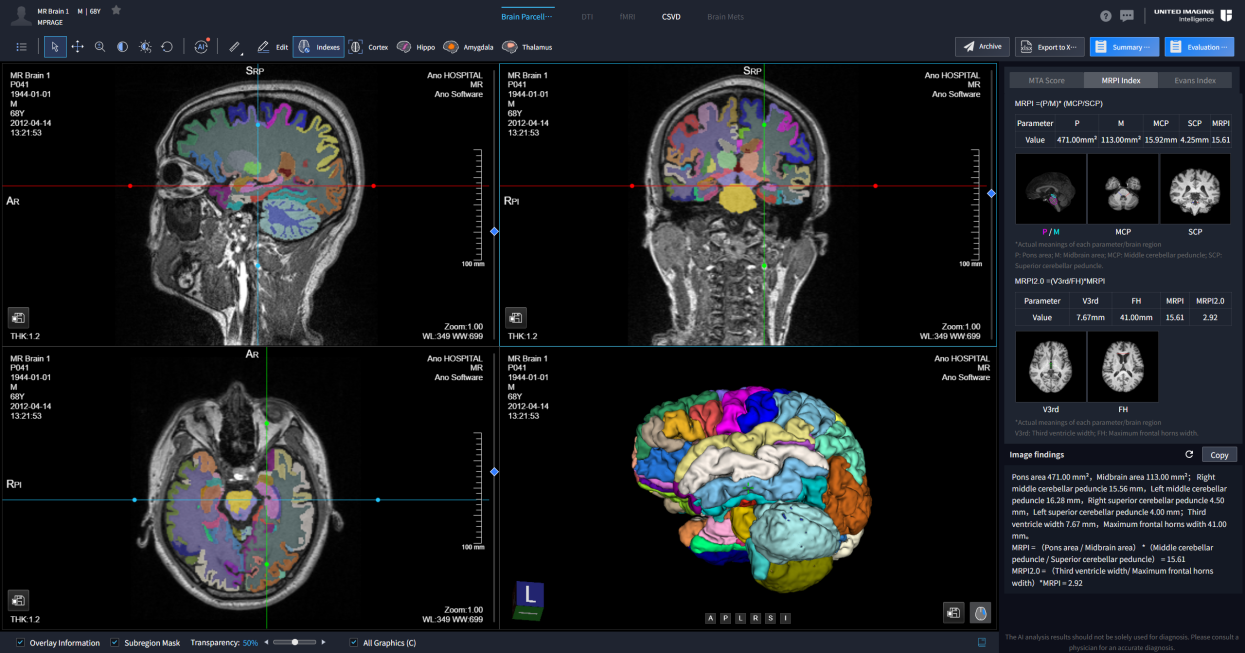


Supplementary Figure 2. Schematic illustration of the user interface of the uAI Brain Health Evaluation Software.

Supplementary Table 1. Five folds comparative analysis of logistic regression, random forest, and support vector machine (SVM) classifiers.

| Model | AUC | AUC_95CI | Accuracy | Sensitivity | Specificity | F1_score |
| --- | --- | --- | --- | --- | --- | --- |
| Logistic | 0.85 | 0.78-0.93 | 0.80 | 0.59 | 0.88 | 0.62 |
| Random Forest | 0.81 | 0.73-0.89 | 0.80 | 0.45 | 0.93 | 0.55 |
| SVM | 0.81 | 0.73-0.89 | 0.80 | 0.45 | 0.93 | 0.55 |


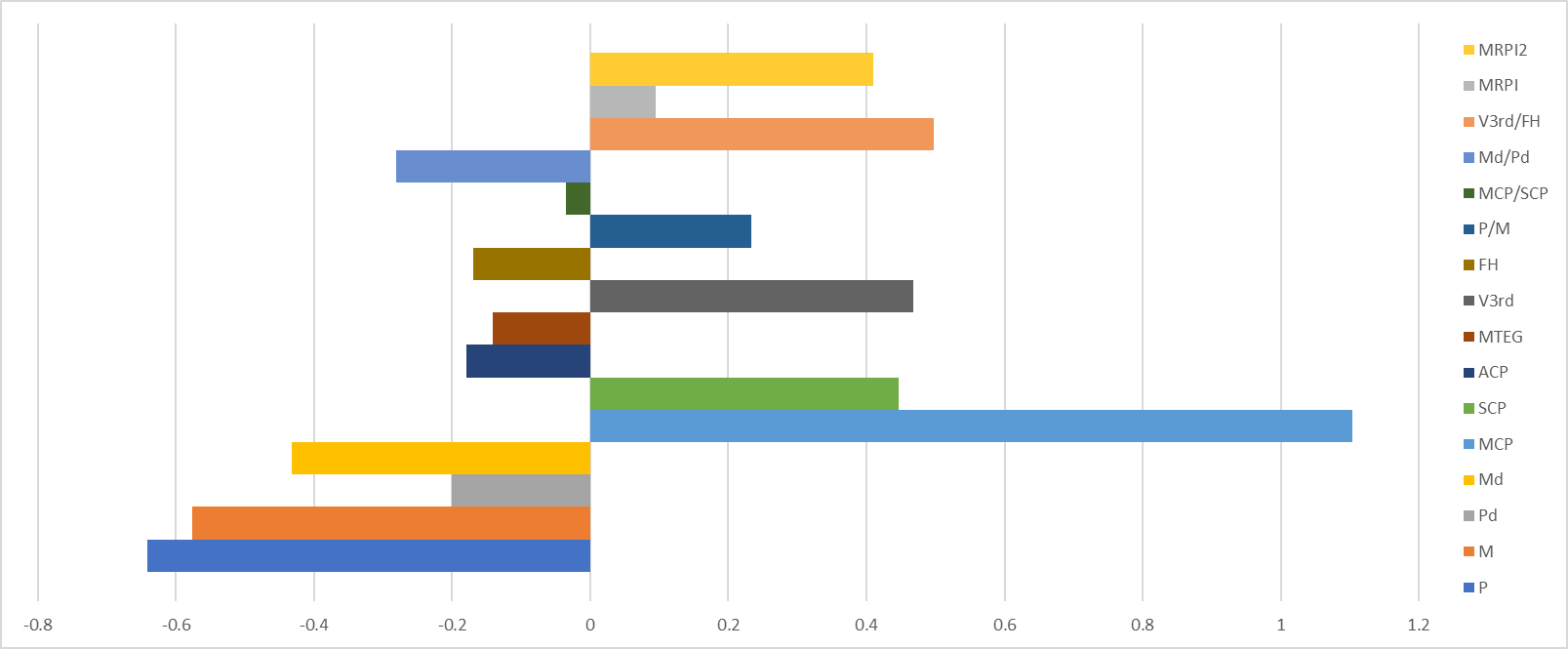


Supplementary Figure 3. Quantitative analysis of feature importance based on the values of the average coefficients from the five-fold cross-validated logistic regression models.

1 Avants, B. B., Tustison, N. & Song, G. Advanced normalization tools (ANTS). *OR Insight* **1–35** (2008).

2 Massey, L. A. *et al.* The midbrain to pons ratio: a simple and specific MRI sign of progressive supranuclear palsy. *Neurology* **80**, 1856-1861, doi:10.1212/WNL.0b013e318292a2d2 (2013).

3 Bradski, G. R. & Kaehler, A. Learning OpenCV - computer vision with the OpenCV library: software that sees. *DBLP* (2008).

4 Quattrone, A. *et al.* A New MRI Measure to Early Differentiate Progressive Supranuclear Palsy From De Novo Parkinson's Disease in Clinical Practice: An International Study. *Mov Disord* **36**, 681-689, doi:10.1002/mds.28364 (2021).

5 Quattrone, A., Antonini, A., Vaillancourt, D. E., Seppi, K. & Quattrone, A. A New MRI Measure to Early Differentiate Progressive Supranuclear Palsy From De Novo Parkinson's Disease in Clinical Practice: An International Study. *Movement Disorders* (2020).

6 Nigro, S. *et al.* Automated MRI Classification in Progressive Supranuclear Palsy: A Large International Cohort Study. *Mov Disord* **35**, 976-983, doi:10.1002/mds.28007 (2020).

7 Wang, D. J., Pandey, S. K., Lee, D. H. & Sharma, M. The Interpeduncular Angle: A Practical and Objective Marker for the Detection and Diagnosis of Intracranial Hypotension on Brain MRI. *American Journal of Neuroradiology* **40** (2019).

8 Quattrone, A. *et al.* A new MR imaging index for differentiation of progressive supranuclear palsy-parkinsonism from Parkinson's disease. *Parkinsonism Relat Disord* **54**, 3-8, doi:10.1016/j.parkreldis.2018.07.016 (2018).

9 Rahul S Desikan, Florent Ségonne, Bruce Fischl, et al., An automated labeling system for subdividing the human cerebral cortex on MRI scans into gyral based regions of interest. NeuroImage 31:3, 968-980, 2006.

10 Xi-Nian Zuo, Jeffrey S Anderson, Pierre Bellec, et al., An open science resource for establishing reliability and reproducibility in functional connectomics. Sci Data 1, 140049, 2014.

11 Dongdong Gu, Feng Shi, Rui Hua, et al., An artificial-intelligence-based age-specific template construction framework for brain structural analysis using magnetic resonance images, Human Brain Mapping. 2022 Oct 21.

12 Rui Hua, Quan Huo, Yaozong Gao, He Sui, Bing Zhang, Yu Sun, Zhanhao Mo, and Feng Shi, Segmenting Brain Tumor using Cascaded V-Nets in Multimodal MR Images, Frontiers in Computational Neuroscience 14:9, 2020.

13 Feng Shi, Weigang Hu, Jiaojiao Wu, et al., "Deep learning empowered volume delineation of whole-body organs-at-risk for accelerated radiotherapy", Nature Communications, 12(1):3242, 2022.
